# Population genetic structure of Marbled Rockfish, *Sebastiscusmarmoratus* (Cuvier, 1829), in the northwestern Pacific Ocean

**DOI:** 10.3897/zookeys.830.30586

**Published:** 2019-03-14

**Authors:** Lu Liu, Xiumei Zhang, Chunhou Li, Hui Zhang, Takashi Yanagimoto, Na Song, Tianxiang Gao

**Affiliations:** 1 The Key Laboratory of Mariculture (Ocean University of China), Ministry of Education, 266071 Qingdao, China Ocean University of China Qingdao China; 2 Laboratory for Marine Fisheries and Aquaculture, Qingdao National Laboratory for Marine Science and Technology, 266072 Qingdao, China Qingdao National Laboratory for Marine Science and Technology Qingdao China; 3 South China Sea Fisheries Research Institute, Chinese Academy of Fishery Sciences, 510003 Guangzhou, China South China Sea Fisheries Research Institute Guangzhou China; 4 Key Laboratory of Marine Ecology and Environment Sciences, Institute of Oceanology, Chinese Academy of Sciences, 266071 Qingdao, China Institute of Oceanology, Chinese Academy of Sciences Qingdao China; 5 National Research Institute of Fisheries Science, Japan Fisheries Research and Education Agency, 2368648 Yokohama, Japan Japan Fisheries Research and Education Agency Yokohama Japan; 6 Fisheries College, Zhejiang Ocean University, 316000 Zhoushan, China Zhejiang Ocean University Zhoushan China

**Keywords:** Genetic diversity, genetic structure, historical population demographics, mtDNA control region, *
Sebastiscus marmoratus
*

## Abstract

*Sebastiscusmarmoratus* is an ovoviviparous fish widely distributed in the northwestern Pacific. To examine the gene flow and test larval dispersal strategy of *S.marmoratus* in Chinese and Japanese coastal waters, 421 specimens were collected from 22 localities across its natural distribution. A 458 base-pair fragment of the mitochondrial DNA (mtDNA) control region was sequenced to examine genetic diversity and population structure. One-hundred-six variable sites defined 166 haplotypes. The populations of *S.marmoratus* showed high haplotype diversity with a range from 0.8587 to 0.9996, indicating a high level of intrapopulation genetic diversity. Low non-significant genetic differentiation was estimated among populations except those of Hyogo, Behai, and Niiigata, which showed significant genetic differences from the other populations. The demographic history examined by neutrality tests, mismatch distribution analysis, and Bayesian skyline analysis suggested a sudden population expansion dating to the late Pleistocene. Recent population expansion in the last glacial period, wide dispersal of larvae by coastal currents, and the homogeneity of the environment may have important influences on the population genetic pattern. Knowledge of genetic diversity and genetic structure will be crucial to establish appropriate fishery management of *S.marmoratus*.

## Introduction

The Marbled Rockfish, *Sebastiscusmarmoratus* (Cuvier, 1829), valued for its high nutritional value and palatability (Zhu et al. 2011), is widely distributed in the coastal areas of the Northern Pacific Ocean, especially in China, Korea, and Japan (Higuchi and Kato 2002; Jin 2006; Nakabo 2013). In recent years, the number of *S.marmoratus* dramatically decreased due to overfishing, pollution, and habitat destruction , potentially influencing its genetic diversity and population structure.

The mitochondrial DNA (mtDNA) control region has been shown to be particularly effective in detecting population genetic structure and diversity, owing to its high polymorphism, maternal inheritance, high mutation rate, and nonrecombinant DNA (Bowen and Grant 1997; Whitehead et al. 2003; Dowling et al. 2008). Zhang et al. (2016) used mtDNA variation to determine that currents and larva dispersion with drifting seaweed influenced the phylogeographic pattern and genetic homogeneity of *Sebastiscusschlegelii* (Hilgendorf, 1880).

While *S.marmoratus* has been widely studied with respect to taxonomy (Hansen and Karlsbakk 2018), genetics (Deng et al. 2015; Cai et al. 2017; Xu et al. 2017), culture (Yin and Qian 2017; Watanabe et al. 2018), and breeding habitat (Chen et al. 2016; Guo et al. 2016), the genetic structure of its populations along the Chinese and Japanese coasts is not known. In view of the many genetic studies based on mtDNA (Zhao et al. 2017; Liu et al. 2018), we selected mtDNA markers to analyse the population genetics of *S.marmoratus*.

The goals of this study were to estimate genetic diversity, to characterize genetic structure, and to reconstruct the evolutionary relationships of *S.marmoratus* in its distribution range. Failure to characterize population units can lead to overfishing and severe decline (Waples 1998). Elucidation of *S.marmoratus* population genetic structure is crucial for its conservation management. The wide distribution of *S.marmoratus* throughout the NW Pacific, along with its short-distance migration life history, makes it an ideal candidate for investigating how the complex geological history of the Northwestern (NW) Pacific shapes intra-species diversity of the fish fauna.

## Materials and methods

### Sample collection

**Figure 1.**
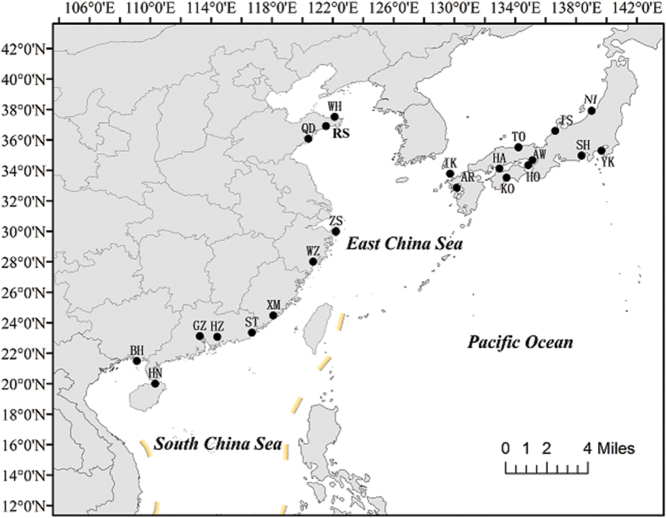
Sampling sites of *S.marmoratus*.

From June 2009 to August 2015, we collected 421 wild *S.marmoratus* from 22 locations in coastal China and Japan, 10–24 specimens per site (Fig. 1, Table 1). Muscle tissue samples were preserved in 95% ethanol for subsequent DNA extraction.

**Table 1.** Sampling information for *S.marmoratus*.

Region	Population	Abbreviation	Number size	Collection date
China coast	Weihai	WH	24	June, 2009
	Rushan	RS	23	June, 2009
	Qingdao	QD	24	July, 2009
	Zhoushan	ZS	24	January, 2015
	Wenzhou	WZ	14	September, 2010
	Xiamen	XM	24	March, 2014
	Shantou	ST	21	August, 2015
	Huizhou	HZ	21	September, 2010
	Guangzhou	GZ	18	September, 2010
	Hainan	HN	14	September, 2010
	Beihai	BH	24	February, 2015
**Total**			**231**	
Japan coast	Niigata	NI	15	June, 2015
	Ishikawa	IS	10	September, 2012
	Yokosuka	YK	22	November, 2011
	Tottori	TO	24	June, 2015
	Shizuoka	SH	12	September, 2012
	Awaji	AW	15	September, 2012
	Hyogo	HO	24	June, 2015
	Hakata Island	HA	14	November, 2011
	Kochi	KO	23	September, 2012
	Iki Island	IK	21	September, 2012
	Ariake-kai	AR	10	September, 2012
**Total**			**190**	

### DNA extraction, amplification, and sequencing

Genomic DNA was extracted from muscle tissue by proteinase K digestion followed by a standard phenol-chloroform technique. Fragments of the mtDNA control region were amplified with primers referenced from Han et al. (2008): DL-S (5’-CCC ACC ACT AAC TCC CAA AGC-3’), DL-R (5’-CTG GAA AGA ACG CCC GGC ATG-3’).

Polymerase chain reactions (PCR) were carried out in 25 μL of reaction mixture containing 10–100 ng template DNA, 0.1 µL (5 U/µL) Taq DNA polymerase (Takara Co., Dalian, China), 1.5 µL (10 pmol/µL) of each forward and reverse primer, and 2 µL (200 µmol/L) deoxy-ribonucleoside triphosphate (dNTP). The PCR amplification was conducted in a Biometra thermal cycler under the following conditions: 2 min initial denaturation at 95 °C; 40 cycles of 60 s at 94 °C for denaturation, 45 s at 52 °C for annealing, and 60 s at 72 °C for extension; and a final extension at 72 °C for 8 min. The PCR product was purified with a Gel Extraction Mini Kit (Watson BioTechnologies Inc., Shanghai, China). The purified product was used as the template DNA for cycle sequencing reactions performed using BigDye Terminator Cycle Sequencing Kit (v. 2.0, PE Biosystems, Foster City, CA, USA), and bi-directional sequencing was conducted on an Applied Biosystems Instrument Prism 3730 automatic sequencer (Sunny Biotechnology Co. Ltd, Shanghai, China) with both forward and reverse primers. The primers used for sequencing were the same as those used for PCR amplification.

### Data analysis

All sequences were edited and aligned manually by DNAStar software (DNAStar Inc., Madison, WI, USA) using default settings and were manually corrected. The genetic diversity indices of *S.marmoratus*, including haplotype diversity (*h*), nucleotide diversity (*π*), mean number of pairwise differences (*k*), and number of polymorphic sites were calculated by ARLEQUIN v. 3.5 (Excoffier and Lischer 2010).

Nucleotide sequence evolution models were evaluated using likelihood-ratio tests implemented by Modeltest v. 3.06 (Posada and Crandall 1998). The neighbor-joining (NJ) tree of the haplotypes was rooted with the out-group *Sebastesschlegelii* (Zhang et al. 2016) using MEGA v.5.0 and evaluated with 1000 bootstrap replicates (Tamura et al. 2011) to reconstruct phylogenies of haplotypes. Among-site heterogeneity was corrected with the shape parameter of gamma distribution (γ = 0.697). The GenBank accession number of *S.schlegelii* is JX241455. Pairwise genetic divergence among populations were tested by the fixation index *F*st (Excoffier et al. 1992), and the significance of the *F*st was evaluated by 10,000 permutations for each pairwise comparison in ARLEQUIN v. 3.5 (Excoffier and Lischer 2010). The *P* values were adjusted by Bonferroni correction (Rice 1989). The *F*st and *P*-value heatmaps with dendrograms were created with the R project 3.5.1 (www.r-project.org).

Characterization of population subdivisions and population structure were conducted using a hierarchical analysis of molecular variance (AMOVA) of different gene pools (Excoffier et al. 1992). In addition to separate total population into the same gene pool analysis, AMOVA analyses were carried out on populations from the Chinese and Japanese coast; North China coast, South China and South Japan coast; North Yellow Sea, South Yellow Sea, East China Sea, South China Sea, and the Japanese coast. The haplotypes were assessed with 1000 permutations in AMOVA.

The Tajima *D* and Fu’s *F*_S_ tests were examined for neutrality (Tajima 1989; Fu 1997). Historical demographic expansions were also investigated by examination of the frequency distribution of pairwise differences between sequences (mismatch distribution) based on three parameters: *θ_0_*, *θ_1_* (*θ* before and after the population growth), and τ (time since expansion expressed in unit of mutational time) (Rogers and Harpending 1992). Historical pure demographic and range expansions were further investigated by the mismatch distributions using ARLEQUIN v. 3.5 (Excoffier and Lischer 2010). Unimodal distribution patterns reflect recent demographic or range expansion with a high level of migration between neighboring demes, while multimodal patterns indicate relatively stationary populations (Rogers and Harpending 1992; Ray et al. 2003). The sum of square deviations (SSD) and Harpending’s raggedness index (HRI) were used to test goodness-of-fit of the observed unimodal mismatch distribution to that expected under the sudden expansion model. The time since population expansion was estimated using the equation τ = 2μt, where μ is the mutation rate for the entire DNA sequence under study, and t is the time since expansion. We used the sequence divergence rate of 5%-10%/MY (Brunner et al. 2001) for the control region sequences.

Bayesian skyline analyses, implemented in BEAST v. 1.7.4 (Drummond and Rambaut 2007), were performed to estimate changes in effective population size through time, which can indicate past demographic changes by comparison with current patterns of genetic diversity within a population (Drummond et al. 2005). To check for convergence, we executed multiple independent runs for 300,000,000 iterations under an HKY+I+G nucleotide substitution model and a strict molecular clock, with individual parameters estimated from the data with a piecewise constant skyline model of 10 groups. Genealogies and model parameters were sampled every 10,000 generations with the first 10% discarded as burn-in. Trace plots were inspected to assess mixing, convergence, and stationary distribution of the MCMC process in Tracer v. 1.5 (Rambaut and Drummond 2009). The effective population sizes were checked and confirmed as >200 for each parameter in order to avoid autocorrelation of parameter sampling.

## Results

### Genetic diversity

A 458 bp segment of the mtDNA control region was amplified, and 106 polymorphic sites were detected, including 89 transitions and 17 transversions. A total of 166 haplotypes were identified based on the sequence variation in 421 individuals from 22 locations. Among these, 84 haplotypes were shared. The most common haplotypes, Hap4 and Hap5, were both shared by 40 individuals. Haplotype sequences were deposited in GenBank under accession numbers KY703229–KY703394.

The estimated nucleotide diversity (*π*) and haplotype diversity (*h*) for the locations are shown in Table 2. The mean value of *π* was 0.0220±0.0110 with highest in Kochi (0.0250±0.0132) and the lowest in Hyogo (0.0098±0.0056). The mean value of *h* was 0.9560±0.0035 with the highest in Awaji (0.9996±0.0243) and the lowest in Hyogo (0.8587±0.0337).

**Table 2.** Genetic diversity parameters among population of *S.marmoratus* from 22 locations.

Population code	Number of haplotypes	Haplotype diversity (*h*)	Nucleotide diversity (*π*)	Number of polymorphic sites (*S*)	Mean number of pairwise (*k*) *differences*
WH	23	0.9964±0.0133	0.0235±0.0123	46	10.753±5.072
RS	14	0.9526±0.0252	0.0216±0.0114	38	9.874±4.689
QD	23	0.9964±0.0133	0.0208±0.0110	38	9.532±4.531
ZS	16	0.9601±0.0238	0.0219±0.0116	36	10.024±4.748
WZ	12	0.978±0.0350	0.0167±0.0093	31	7.634±3.789
XM	18	0.9565±0.0311	0.0236±0.01244	42	10.795±5.090
ST	15	0.9524±0.0317	0.0194±0.0100	36	8.090±4.269
HZ	14	0.9524±0.0278	0.0173±0.0093	27	7.884±3.820
GZ	16	0.9804±0.0284	0.0221±0.0118	36	10.111±4.847
HN	13	0.9890±0.0314	0.0110±0.0063	26	9.050±4.435
BH	16	0.9638±0.0208	0.0140±0.0077	32	6.408±3.145
AR	9	0.9778±0.0540	0.0216±0.0122	31	9.878±4.943
IK	15	0.9571±0.0301	0.0239±0.0126	39	10.940±5.184
NI	10	0.9238±0.0530	0.0156±0.0089	30	7.130±3.544
IS	7	0.8667±0.1072	0.0224±0.0126	25	10.251±5.117
TO	19	0.9783±0.0187	0.0224±0.0118	39	10.275±4.859
SH	11	0.9848±0.0403	0.0224±0.0124	28	10.242±5.033
YK	19	0.9740±0.0276	0.0229±0.0121	45	10.471±4.963
KO	14	0.9368±0.0306	0.0251±0.0132	45	11.485±5.404
AW	15	0.9996±0.0243	0.0171±0.0095	26	7.846±3.869
HA	10	0.9560±0.0377	0.0179±0.0099	28	8.209±4.052
HO	7	0.8587±0.0337	0.0098±0.0056	13	4.520±2.304
Total	166	0.956±0.0035	0.022±0.011	106	9.952±4.561

### Population structure

**Figure 2.**
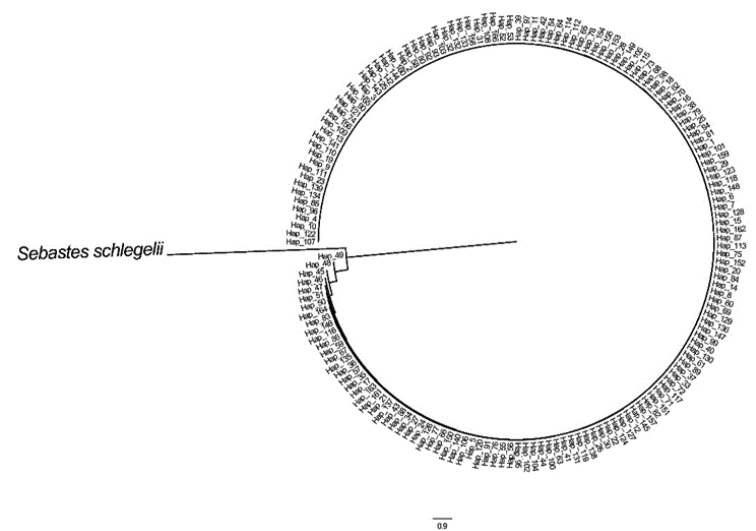
Phylogenetic tree of control region haplotypes constructed using neighbor-joining algorithms of *S.marmoratus* with *S.schlegelii* as outgroup.

**Figure 3.**
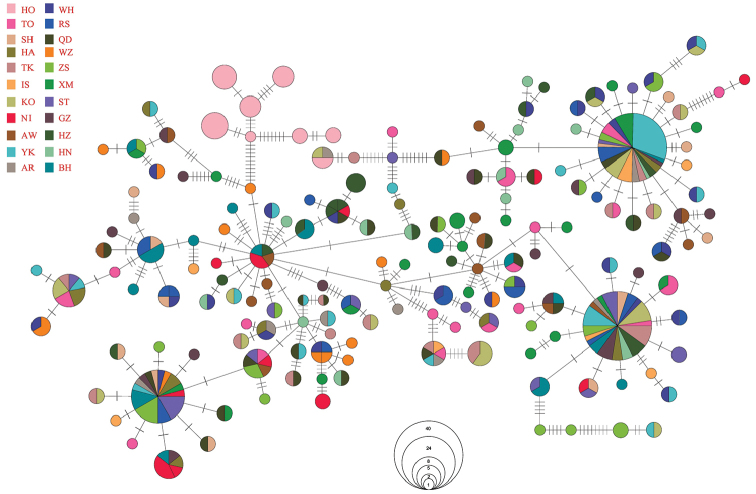
Median-joining network of *S.marmoratus* haplotypes.

An unrooted phylogenetic tree was reconstructed by neighbor-joining analysis using 166 haplotypes with the best nucleotide substitution mode (HKY+I+G) rooted with the outgroup *S.schlegelii*. There were no significant genealogical branches or clusters corresponding to sampling localities (Fig. 2). The relationships among 166 haplotypes were represented on the minimum spanning tree (MST). The minimum spanning network was generally star-like with several common and ancestral haplotypes shared by most populations (Fig. 3). The MST was connected and indicated recent population expansion. The Hyogo population showed an obvious haplotype branch and others exhibited no unique haplotype corresponding to geographic populations.

Genetic differentiation among the 22 locations was evaluated based on *F*st values (Table 3, Fig. 5) and AMOVA analyses (Table 4). In general, most of the pairwise *F*st values among populations showed non-significant differences after sequential Bonferroni correction. However, significant genetic differences were obtained among Hyogo, Behai, and Niigata populations and between Hyogo, Behai, and Niigata and the other populations. The largest difference was seen between Niigata and Hyogo (*F*st = 0.677, *P* < 0.05). Some pairwise *F*st estimates were negative, indicating that within-population variation was greater than that between populations. The global AMOVA showed about 13.59% of the genetic variation to be among populations. Other grouping methods also indicated most genetic variation to be within populations, rather than among groups and populations.

**Table 3.** Pair-wise *F*_ST_ (below diagonal) sampling locations of *S.marmoratus*.

	**BH**	**GZ**	**HA**	**HN**	**HO**	**HZ**	**IK**	**IS**	**KO**	**QD**	**RS**	**SH**	**ST**	**TO**	**WH**	**WZ**	**XM**	**NI**	**YK**	**ZS**	**AR**	**AW**
BH																						
GZ	0.034																					
HA	0.006	-0.023																				
HN	0.147*	0.004	0.026																			
HO	0.674*	0.610*	0.657*	0.654*																		
HZ	0.085*	0.018	0.017	-0.001	0.649*																	
IK	0.081*	-0.003	-0.002	-0.001	0.611*	0.035																
IS	0.232*	0.036	0.096	-0.022	0.674*	0.079	0.012															
KO	0.143*	0.021	0.033	-0.022	0.604*	0.044	-0.023	-0.034														
QD	0.057*	-0.016	-0.020	-0.016	0.613*	0.001	0.003	0.028	0.018													
RS	0.054*	-0.014	-0.020	-0.011	0.613*	0.003	-0.005	0.027	0.005	-0.016												
SH	0.019	-0.036	-0.054	-0.007	0.633*	0.011	-0.008	0.037	0.010	-0.033	-0.039											
ST	-0.005	-0.015	-0.030	0.053	0.638*	0.038	0.017	0.107	0.059*	0.004*	0.003	-0.026										
TO	0.084*	-0.009	-0.004	-0.005	0.609*	0.028	-0.017	-0.005	-0.013	-0.012	-0.010	-0.017	0.020									
WH	0.092*	-0.007	0.004	-0.034	0.599*	-0.004	0.007	0.001	-0.003	-0.013	0.026	-0.017	0.027*	-0.009								
WZ	0.041	0.046	-0.018	0.109	0.664*	0.077	0.064*	0.200*	0.109*	0.042*	0.050	0.024	0.036	0.074	0.067							
XM	0.096*	-0.002	0.011	-0.018	0.602*	0.019	0.004	-0.016	-0.001	-0.064	-0.010	-0.012	0.021*	-0.008	-0.020	0.081*						
NI	0.081*	0.066	0.012	0.116	0.677*	0.094	0.100*	0.211*	0.136*	0.051*	0.083	0.042*	0.056	0.110	0.101	0.030	0.103*					
YK	0.034	-0.018	-0.029	0.005	0.608*	0.014	-0.012	0.042	0.007	-0.013	-0.009	-0.022	-0.011	-0.017	-0.012	0.030	-0.002	0.070*				
ZS	0.061*	-0.002	-0.016	0.015	0.610*	0.033	0.015	0.042	0.023	0.009	0.007	-0.016	-0.001	0.009	0.010	0.045	0.004	0.038	-0.010			
AR	0.030	-0.031	-0.060	-0.013	0.650*	0.006	-0.052	0.026	-0.016	-0.038	-0.038	-0.049	-0.020	-0.036	-0.022	0.002	-0.016	0.004	-0.047	-0.023		
AW	0.026	-0.029	-0.019	0.020	0.655*	0.010	0.012	0.074	0.033	-0.012	-0.023	-0.029	-0.070	0.002	-0.012	0.051	-0.010	0.097*	-0.020	0.008	0.029	

**Table 4.** AMOVA of *S.marmoratus* populations based on mtDNA control region sequences.

Source of variation	Observed partition			
	Variance components	Percentage variation	Φ Statistics	*P*
1. *Complete gene pool (WH, RS, QD, ZS, WZ, XM, ST, HZ, GZ, HN, BH, AR, IK, NI, IS, TO, SH, YK, KO, AW, HA, HO)*				
Among populations	0.6804	13.59	Φ_ST_=0.1359	0.0000±0.0000
Within populations	4.3268	86.41		
2. *Two gene pools (WH, RS, QD, ZS, WZ, XM, ST, HZ, GZ, HN, BH) (AR, IK, NI, IS, TO, SH, YK, KO, AW, HA, HO)*				
Among groups	0.0224	0.45	Φ_CT_=0.0045	0.1927±0.0038
Among populations within groups	0.6688	13.33	Φ_SC_=0.1339	0.0000±0.0000
Within populations	4.3269	86.23	Φ_ST_=0.1377	0.0000±0.0000
3. *Three gene pools (WH, RS, QD) (ZS, WZ, XM, ST, HZ, GZ, HN, BH) (AR, IK, NI, IS, TO, SH, YK, KO, AW, HA, HO)*				
Among groups	-0.0389	-0.78	Φ_CT_=-0.0078	0.6663±0.0045
Among populations within groups	0.7059	14.14	Φ_SC_=0.1403	0.0000±0.0000
Within populations	4.3268	86.64	Φ_ST_=0.1336	0.0000±0.0000
4. Five gene pools (*WH*) (*RS, QD*) (*ZS, WZ*) (*XM, ST, HZ, GZ, HN, BH) (AR, IK, NI, IS, TO, SH, YK, KO, AW, HA, HO)*				
Among groups	-0.1703	-3.43	Φ_CT_=-0.8840	0.8841±0.0032
Among populations within groups	0.8066	16.25	Φ_SC_=0.0000	0.0000±0.0000
Within populations	4.3269	87.18	Φ_ST_=0.0000	0.0000±0.0000

### Population historical demography

**Figure 4.**
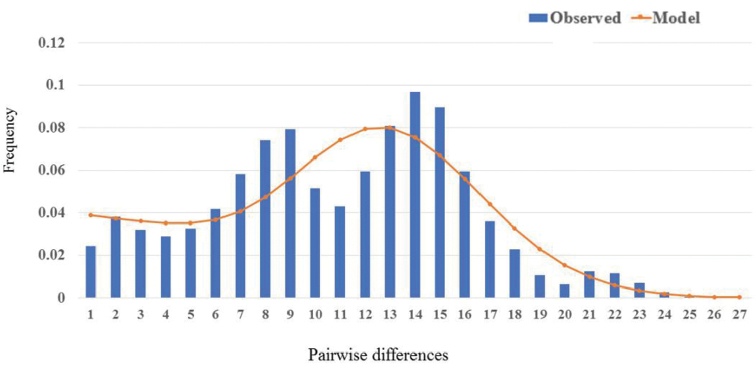
The observed pairwise difference (bars) and the expected mismatch distributions under the sudden-expansion model (solid line) of mtDNA control region haplotypes in *S.marmoratus*.

Tajima’s *D* (*D* = -1.027; *P* > 0.05) and *F*s test (*F*s = -23.917; *P* < 0.01) results were negative, indicating departure from selective neutrality (Table 5). Non-significant and low values of SSD and HRI were found for each population and for the overall population, suggesting a sudden expansion model. The sudden expansion model of mismatch distribution was unimodal and a valid goodness-of-fit was observed between observed and expected distributions (Fig. 4), indicating strong demographic expansion. The τ value, which reflects the location of the mismatch distribution crest, provides a rough estimate of the time of initiation of rapid population expansion. According to *t* = *τ/2μ*, based on τ values (τ = 12.305) and divergence rate of 5–10% per site per Myr (Brunner et al. 2001), the pure population expansion occurred 268,000–448,000 years ago. The ratio of estimated effective female population size after expansion to that before expansion (*θ_1_/θ_0_*) was 56.84 (Table 4). Results indicated that *S.marmoratus* underwent colonization and recent population expansion events along the Chinese and Japanese coasts during the Pleistocene.

The Bayesian skyline plot (Fig. 6) indicated an historic occurrence of a continual gradual increase in the effective size of *S.marmoratus* populations, dating to about 430,000 years BP at the end of the Pleistocene. These results are consistent with a process of historical expansion of *S.marmoratus* populations, as indicated by negative *D* and *F*s values and mismatch distributions.

**Figure 5.**
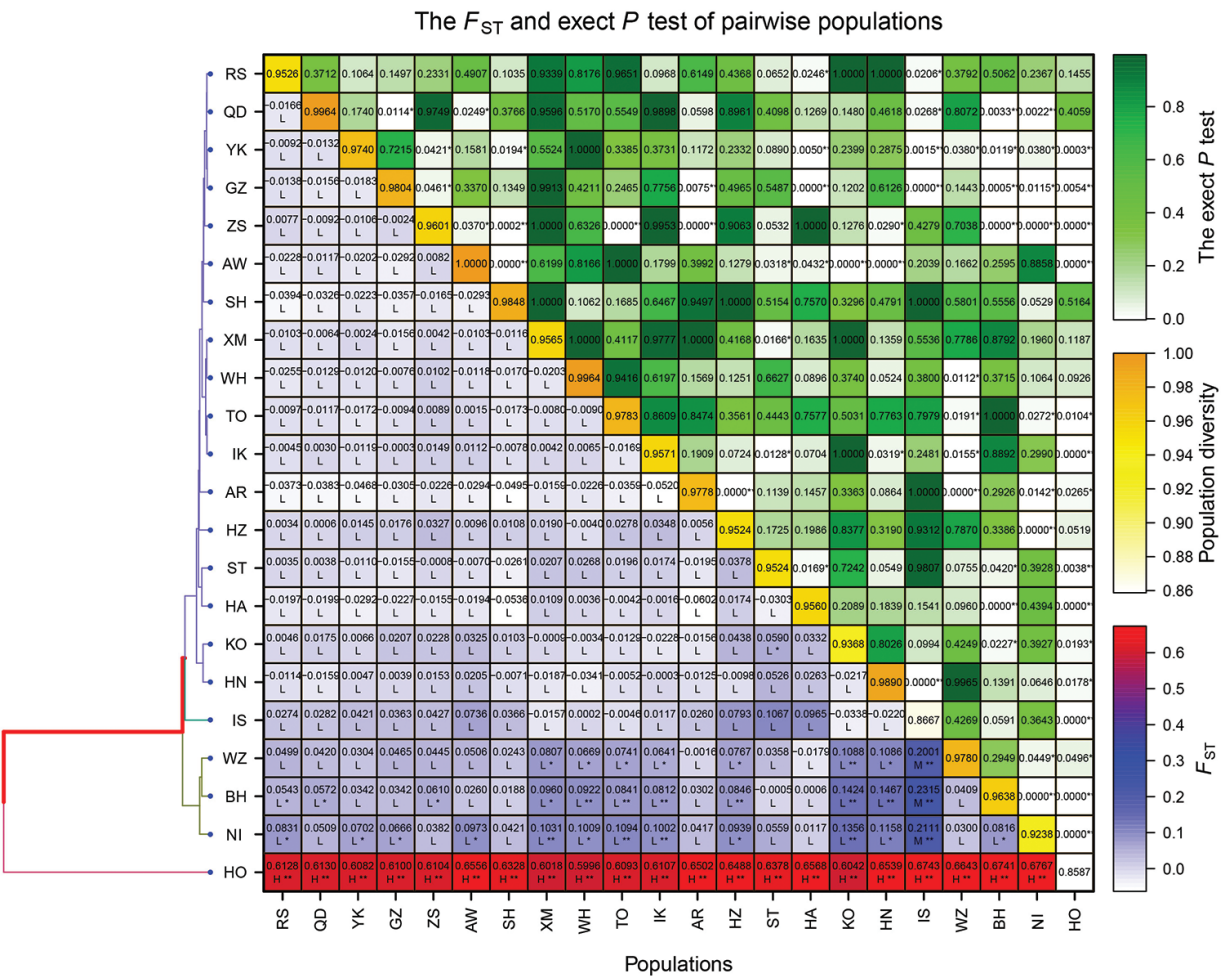
The heatmap of *F*_st_ genetic distances based on mtDNA control sequences of populations.

**Figure 6.**
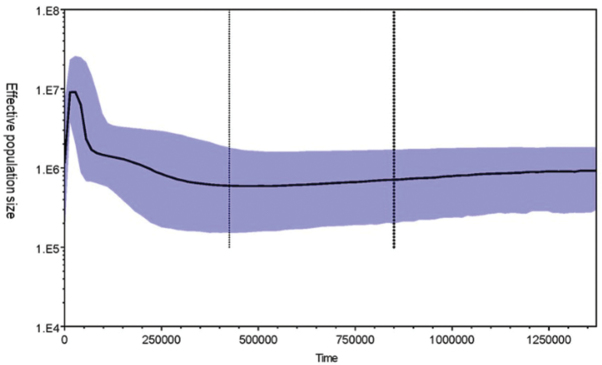
Bayesian skyline plot showing the effective female *S.marmoratus* population size through time. Black solid lines are median estimates of NeT (Ne=effective female population size; T=generation time); blue shading represents the 95% confidence interval of NeT. The y-axis was plotted on a logarithmic scale.

**Table 5.** Tajima’s *D* and Fu’s *F_S_*, corresponding *P*-value, and mismatch distribution parameter estimates for each population of *S.marmoratus*.

Population	Tajima’s *D*		Fu’s *F*s		Mismatch distribution				
	*D*	*P*	*Fs*	*P*	*τ* (95% CI)	*θ_0_*	*θ_1_*	*SSD*	*HRI*
BH	-1.089	0.149	-4.828	0.031	6.709 (4.043,10.332)	0.002	26.364	0.009ns	0.480ns
GZ	-0.291	0.442	-5.588	0.016	12.738 (5.533, 16.066)	0.000	48.730	0.018ns	0.027ns
HA	-0.508	0.333	-1.114	0.271	8.049(4.088,10.371)	0.009	66.982	0.058ns	0.124ns
HN	0.177	0.615	-4.933	0.015	12.803(5.627,19.664)	0.002	23.016	0.020ns	0.039ns
HO	0.878	0.832	1.688	0.782	5.467(0.803,10.469)	0.967	9.946	0.032ns	0.069ns
HZ	-0.034	0.542	-2.647	0.120	13.922(0.000,85.547)	0.000	13.865	0.011ns	0.016ns
IK	-0.224	0.450	-2.256	0.173	12.078(7.684,14.979)	0.000	99.219	0.023ns	0.051ns
IS	0.363	0.694	0.757	0.628	14.285(6.186,20.066)	0.005	29.102	0.046ns	0.084ns
KO	-0.405	0.372	-0.645	0.402	13.199(8.232,17.143)	0.000	83.906	0.032ns	0.060ns
QD	-0.457	0.351	-14.813	0.000	11.434(4.629,16.014)	0.139	29.270	0.016ns	0.022ns
RS	-0.404	0.369	-1.192	0.314	13.385(4.031,18.676)	0.002	23.507	0.034ns	0.035ns
SH	0.118	0.592	-3.022	0.050	8.398(1.467,91.398)	4.888	38.945	0.046ns	0.050ns
ST	-0.659	0.297	-3.169	0.092	6.348(1.977,22.352)	4.104	31.631	0.034ns	0.036ns
TO	-0.215	0.474	-5.824	0.025	11.219(5.793,13.742)	0.014	54.141	0.003ns	0.007ns
WH	-0.721	0.246	-13.615	0.000	13.646(5.686,18.615)	0.004	27.832	0.012ns	0.011ns
WZ	-1.115	0.133	-3.865	0.030	9.010(4.488,12.814)	0.021	24.199	0.010ns	0.030ns
XM	-0.417	0.384	-4.241	0.063	14.230(7.807,18.992)	0.000	33.264	0.025ns	0.026ns
NI	-1.142	0.116	-1.163	0.259	3.281(0.191,19.438)	4.706	18.687	0.028ns	0.064ns
YK	-0.743	0.242	-7.208	0.006	7.867(4.506,16.061)	3.841	90.938	0.018ns	0.023ns
ZS	-0.109	0.527	-2.516	0.174	13.576(5.086,18.025)	0.000	25.162	0.012ns	0.013ns
AR	-0.742	0.240	1.813	0.137	12.568(6.381,16.771)	0.004	47.383	0.029ns	0.061ns
AW	-0.151	0.478	-9.125	0.001	2.242(0.559,13.279)	7.604	99999	0.009ns	0.014ns
Pooled	-1.027	0.133	-23.917	0.005	12.305(8.693,15.988)	0.434	24.668	0.004ns	0.005ns

τ, time of initiation of population expansion, *θ_0_* and *θ_1_* are *θ* parameter before and after expansion, *SSD* and *HRI* are sum of squared deviations and raggedness index, respectively. *P* > 0.05

## Discussion

Inbreeding depression and other genetic problems impacted by human behavior can be monitored by assessing genetic diversity under natural conditions (Ryman 1991; Smith et al. 1991). The adaption of marine organisms to their surroundings and their evolutionary potential can be affected by genetic diversity (Templeton 2010). We found that, despite high haplotype diversity (*h* = 0.9560±0.0035) of *S.marmoratus* in the northwestern Pacific Ocean, its nucleotide diversity was low (*π* = 0.0220±0.0110). The high mutation rate of the D-loop region may be a factor in this phenomenon (Wan et al. 2004). Haplotype diversity with low nucleotide diversity may indicate population reduction or the existence of a genetic bottleneck and may result in extinction under environmental pressure. It can be observed in a population experiencing rapid expansion from a low effective population size, assuming adequate time for the increase in haplotypes through mutation but inadequate time for accumulation of large sequence differences (Lowe et al. 2004). The retention of new mutations in the population can be enhanced by rapid population growth (Grant and Bowen 1998). The phenomenon of high haplotype diversity and low nucleotide diversity has been reported in organisms such as *Glyptocephalusstelleri* (Xiao et al. 2010), *Trachurusjaponicus* (Song et al. 2013) and *Circusspilonotus* (Nagai et al. 2018) that have undergone a rapid severe population reduction.

Compared with anadromous and freshwater fishes, marine species are generally expected to show a low degree of genetic differences among geographic regions owing to their high dispersal potential through planktonic drifting of eggs, larvae, or adults and the absence of physical barriers (Palumbi 1994; Hewitt 2000; Liu et al. 2018). Our AMOVA results and the neighbor-joining analysis did not show significant genetic structure among geographic populations. Ecological characteristics and marine currents may play important roles in shaping the contemporary phylogeographic pattern of marine fishes. For example, the rockfish *S.schlegelii* is typical of fish that congregate in drifting seaweed during early development (Ikehara 1977; Safran and Omori 1990; Zhang et al. 2016). *Sebastiscusmarmoratus*, a species of rockfish with life history similar to *S.schlegelii*, is believed to exhibit the same behavior, dispersing with drifting seaweed during November and December and in the following year from February to April (Mitchell and Hunter 1970; Wu et al. 1999). The Kuroshio Current is one of the strongest currents in the world and can accelerate gene flow from the southern East China Sea to the coastal waters of Japan (Liu et al. 2007). Inflows from the Yellow Sea enter the Bohai Sea along the west coast of Korea via the Yellow Sea warm current and the China Coastal Current (Jin et al. 2010). Waters also exchange between the warm Yellow Sea and Kuroshio currents. These strong currents might transport *S.marmoratus* larvae via drifting seaweed and promote exchange throughout its range.

Recent research reveals that the currently most common unintentional pathway for the transport of marine organisms is the ballast water of commercial vessels (Ruiz and Hines 1997; Wonham et al. 2000). As human activity becomes more frequent and extensive, trade between countries is strengthened, and commercial vessels traverse large area. Ballast water is usually taken from the harbor in one port and subsequently discharged in another port (Carlton and Geller 1993). Diverse organisms including protist, diatoms, invertebrate larvae, and copepods are collected and survive the voyage to the next port (Carlton 1985; Smith et al. 1999). Corrosion or other damage to protective grates or ballasting of water by gravitation, may provide access to the ballast for larger organisms such as post-larval fish (Springer and Gomon 1975; Wonham et al. 2000). Hansen and Karlsbakk (2018) reported *S.marmoratus* caught by bait in Norwegian waters and thus shown to be actively foraging, a strong indication that *S.marmoratus* may thrive in unfamiliar conditions.

We may conclude that transportation via ballast water may be a source of genetic homogeneity of *S.marmoratus*. It has been transported among ports and wharves along the NW Pacific Ocean and into rocky coastal areas with the release of ballast water, where it can easily survive (Hansen and Karlsbakk 2018). With adaptation to the same or similar environment, a number of invasive populations of *S.marmoratus* have been reported (Wonham et al. 2000). It is also possible that environmental factors such as salinity and temperature have brought about adaptive evolution of *S.marmoratus*. However, this is undetectable by the molecular markers we used in this study. It has been suggested that a genotyping-by-sequencing technique could reveal the occurrence of local adaptation (Xu et al. 2017).

Using genetics to understand biogeography is important to determine patterns influencing distribution of geographically distant populations. Genetic diversity, genetic distribution patterns, and effective population size were also influenced by paleogeological changes and fluctuations as well as life history and marine environment factors (Hewitt 1996). Following sea level falls in the glacial period (Wang 1999; Lambeck et al. 2002), *S.marmoratus* may have experienced a population contraction with the loss of genes of those dying out and the majority of survivors migrating to more suitable environments. A single branch from the star-like network representing the Hyogo population suggests a likely founder effect (Ramachandran et al. 2005).

Significant genetic differences were revealed between Hyogo and other populations based on the star-like network tree and *F*st value analysis, which suggests that the deep and semi-open area of inland waters might have an impact on the geographic isolation. Genetic differences among Hyogo, Behai, and Niigata populations and between Hyogo, Behai, and Niigata and the other populations were primarily significant, and possibly relate to convergence evolution (Wilkens and Strecker 2003) and the formation of a refuge (Consuegra et al. 2002). In addition, mutation-drift disequilibrium may exist among these populations, which are in an unstable state of genetic mutation-drift (Lacy 1987). Further molecular marker studies are required to evaluate this proposition.

## Conclusions

Climate fluctuations caused by glacial-interglacial alternation, early life-history, and ecological characteristics, combined with transport via ballast water may play important roles in the extensive gene flow among populations and the current genetic distribution pattern of *S.marmoratus*. Information provided by the current study will facilitate its comprehensive management. Future studies should be based on informative nuclear markers to provide additional information on genetic structure and differentiation of populations of *S.marmoratus*.
